# Improvement of muscular atrophy by AAV–SaCas9-mediated myostatin gene editing in aged mice

**DOI:** 10.1038/s41417-020-0178-7

**Published:** 2020-05-13

**Authors:** Shaoting Weng, Feng Gao, Juan Wang, Xingyu Li, Beibei Chu, Jiang Wang, Guoyu Yang

**Affiliations:** College of Animal Sciences and Veterinary Medicine, Henan Agricultural University, 450002 Zhengzhou, Henan Province People’s Republic of China

**Keywords:** Genetic engineering, Gene regulation

## Abstract

Muscle mass and area usually decrease with age, and this phenomenon is known as sarcopenia. This age-related atrophy correlates with insufficient levels of muscle cells differentiate and proliferate regulated by the TGF-β signaling pathway and the expression of E3s ubiquitin-protein ligase by the aged. Sarcopenia makes a huge impact on the aging society, because it has the characteristic of high incidence, extensive adverse effects and disease aggravation gradually. Guided by a single-guide RNA (sgRNA), Cas9 nuclease has been widely used in genome editing, opening up a new pathway for sarcopenia treatment. Here, we present two rAAV9 systems, pX601-AAV-CMV:SaCas9-U6:sgRNA and pX601-AAV-EF1α:SaCas9-tRNA_GLN_: sgRNA, which edited myostatin efficiently. By delivering the two rAAV–SaCas9 targets to myostatin via intramuscular injection of aged mice, an increase in body weight and an increase in the number and area of myofibers were observed. Knockout of myostatin led to TGF-β signaling pathway changes, and increased MyoD, Pax7 and MyoG protein levels and increased the number of satellite cells to improve muscle cells differentiation. Moreover, knockout of myostatin prevented the atrophy of muscle cells through reduced Murf1 and MAFbx protein levels. We found that both rAAV–SaCas9 systems had gene editing efficiency, reducing the expression of myostatin by affecting the relevant signaling pathways, thereby altering the physiological status. We showed that myostatin has an important role in activating skeletal muscle proliferation and inhibiting muscular atrophy during aging. Thus, we propose that knockout of myostatin using the rAAV9–SaCas9 system has significant therapeutic potential in sarcopenia.

## Introduction

Cas9 is an RNA-guided endonuclease derived from the type II CRISPR–Cas adaptive immune system of bacterial adaptive immune system. Because of its small size, strong targeting and high editing efficiency, it has been widely used in genome editing and has a broad application prospect in disease therapy. However, genome editing of tissue in post-natal animals is restricted because of the challenge of delivering Cas9 in vivo. In recent years, recombinant adeno-associated virus (rAAV) as a promising gene delivery vector, has been widely used in genome editing at the animal level. The application of this vector in gene therapy possesses several advantages, including extensive serotype specificity, a low oncogenic risk of host-genome integration and low immunogenicity. However, the restrictive cargo size (~4.5 kb) of AAV limit the ability to packaging the commonly used *Streptococcus pyogenes* Cas9 (SpCas9, ~4.2 kb) and its single-guide RNA (sgRNA) into a single vector. The compact SaCas9 (~3.3 kb) which is derived from *Staphylococcus aureus*, is smaller and possesses a more specific protospacer adjacent motif (PAM) than SpCas9, which makes it suitable for delivery using AAV vector. Ran et al. [1] reported that SaCas9 can edit the genome with efficiencies similar to those of SpCas9, and packaged SaCas9 and its sgRNA into a single AAV vector, thus significantly reducing expression level of cholesterol by knocked out Pcsk9 in the mouse liver. El Refaey et al. [2] showed that a single systemic AAV–SaCas9 could effectively excise the mutation on exon23 for dystrophin expression and cardiac function therapy in mdx/Utr^+/^^−^ mice. As the standard AAV–SaCas9 system is almost at its cargo limit, to allow extra space for more sgRNA cassettes and to improve the packaging efficiency, the total length of the SaCas9 system needs to be reduced. Mefferd et al. [3] reported recently that expression of sgRNAs can be driven by small tRNA promoters (~70 bp), which is equivalent to the size of about one half sgRNA-expressing cassette with an U6 promoter. For the efficient delivery of CRISPR materials into cells in vivo, Wei et al. [4] reconstructed the new vector lentiSaCRISPR v2, which was the original lentiCRISPR v2 that targets SpCas9 [5] with the Cas9 and sgRNA cassettes switched to make it adaptable for SaCas9 targeting. Here we generated two versions of SaCas9 constructs with different sgRNA promoters, U6 pro (~241 bp) and tRNA GLN pro (~72 bp), and different SaCas9 promoters, CMV pro (~584 bp) and EF1α pro (~212 bp). These promoters are reported to be able to effectively drive the expression of Cas9 and can be used to efficiently edit genomic DNA in vitro and in vivo.

As a cytokine belonging to the transforming growth factor-beta (TGF-β) superfamily and an effective inhibitor of muscle growth, myostatin (MSTN) is expressed in both developing and adult skeletal muscle, thereby suggesting that MSTN has a sustained role in myogenesis. MSTN-deficient animals indeed show greater muscle mass due to muscle proliferation and hypertrophy [6]. Systemic administration of MSTN leads to decreased skeletal muscle mass in mice. Whereas without MSTN, mice exhibit a 2–3-fold increase in skeletal muscle weight [7]. In humans, a mutation that may lead to mis-splicing of the *Mstn* gene was found in a child with muscle hypertrophy [8]. Siriett et al. [9] showed that MSTN affects myogenesis in mice by regulating myogenic regulators. Carlson et al. showed that TGF-β signaling pathway had an important role in regulating the differentiation and proliferation of muscle cells. It affected satellite cell activation, myoblast differentiation and muscle fiber fusion [10]. It has been reported that enhanced TGF-β signaling pathway impeded muscle regeneration and was an important cause of aged-related muscular atrophy [11]. Wei et al. [12] demonstrated disruption of *Mstn* gene in a few myotubes decreased myostatin expression, resulting in attenuated TGF-β signaling pathways in targeted cells and adjacent cells, thereby reducing the atrophy of these myotubes. Furthermore, MSTN signaled satellite cell quiescence and negatively regulated satellite cell self-renewal [13]. This may have important consequences in age-related muscle atrophy and regeneration. Therefore, we believe that *Mstn* is a promising target for inhibiting skeletal muscle proliferation and activating muscular atrophy, and *Mstn* gene knockout may effectively reduce the loss of skeletal muscle mass in sarcopenia.

To determine the knockout ability of the SaCas9 system and to clarify the effect of *Mstn* on muscle atrophy, this study used both SaCas9 vectors to determine knockout efficiency, and then packaged these vectors into rAAV9 viruses. These two rAAV–SaCas9 viruses were intramuscularly injected in the left thigh of aged C57BL/10 male mice to verify the knockout capacity of *Mstn* in vivo and the effect on cytokines associated with skeletal muscle during age-related atrophy.

## Materials and methods

### Cells

The HEK293 and C2C12 cell lines were purchased from the cell bank of China Committee for Typical Culture Collection, China Academy of Sciences. Cells were tested for mycoplasma contamination and were grown in high-glucose DMEM (Dulbecco’s Modified Eagle Medium) supplemented with 10% FBS (fetal bovine serum), and cultured at 37 °C in a humidified atmosphere of 5% CO_2_.

### Construction of SaCas9 and sgRNA plasmids

**Fig. 1:**
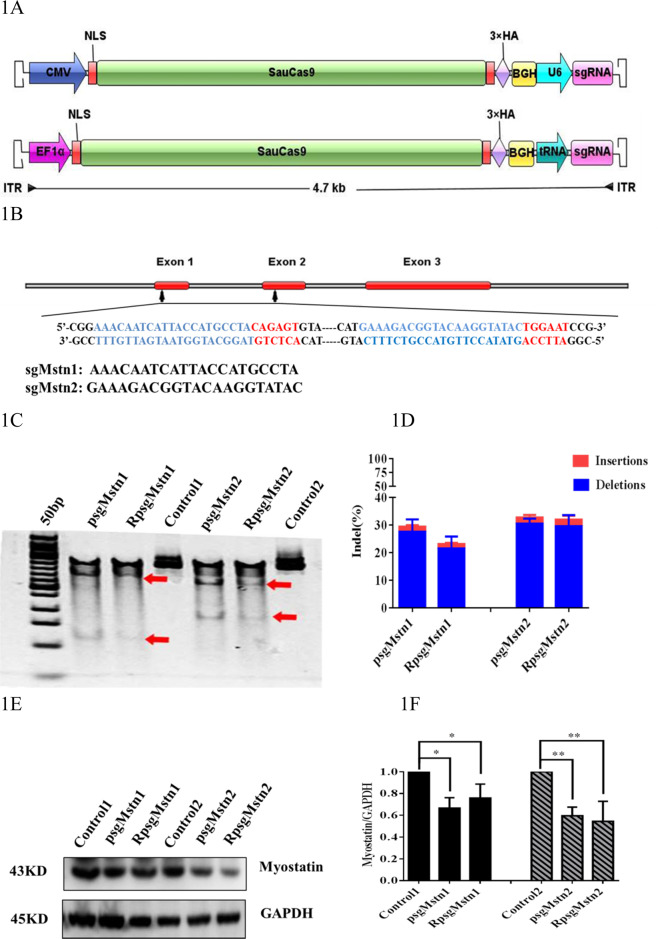
Construct of all-in-one AAV–SaCas9–sgRNA. **a** Schematic representation of two rAAV viruses expressing SaCas9 and its sgRNA with different promoters. The backbone is flanked by AAV inverted terminal repeats (ITR). The poly(A) signal is from rabbit beta-globin (BGH). **b** Schematic diagram of the Myostatin in mice genes. Red bars represent exons. Zoomed-in views show the protospacer sequence (black), whereas the SaCas9 PAM sequence is highlighted in red. Double-stranded break location sites are denoted in blue. **c** T7EI assay of C2C12 genes produced by the transfected cells and the untransfected cells. The transfected cells were treated by psgMstn1, RpsgMstn1, psgMstn2 and RpsgMstn2. The untransfected cells served as controls. Numbers of mutant cells with bands of 133, 379 and 512 bp in psgMstn1, RpsgMstn1 and only one band of 512 bp in control 1. Numbers of mutant cells with bands of 168, 338 and 506 bp in psgMstn2, RpsgMstn2 and only one band of 506 bp in control 2. The red arrow indicates the band of enzyme digestion. **d** Stacked histogram showing the percentage distribution of indels at sgMstn1 and sgMstn2 in C2C12 mutant cells, as measured by sequencing analyses. Data represent means ± SEM from three technical replicates. **e** The C2C12 mutant cells at sgMstn1, sgMstn2 and control analyzed by western blot using an anti-MSTN antibody. And anti-GAPDH was used as loading control. **f** Stacked histogram showing the percentage distribution of MSTN expression in different C2C12 cells. Data represent means ± SEM from three technical replicates. *: 0.01 < *P* < 0.05, **P < 0.01 when psgMstn1, RpsgMstn1-treated or psgMstn2, RpsgMstn2-treated were compared with untreated.

All plasmids were constructed using standard recombinant DNA cloning techniques. pX601-CMV:SaCas9-U6:sgRNA (pX601-AAV-CMV::NLS-SaCas9-NLS-3xHA-bGHpA;U6::*Bsa*I-sgRNA) was preserved in our laboratory. tRNA_GLN_ and sgRNA scaffold sequences were generated by Sangon (Shanghai, China) (GGTTCCATGGTGTAATGGTTAGCACTCTGGACTCTGAATCCAGCGATCCGAGTTCAAATCTCGGTGGAACCT-GAAACACCGGAGACCACGGCAGGTCTCAGTTTTAGTACTCTGGAAACAGAATCTACTAAAACAAGGCAAAATGCCGTGTTTATCTCGTCAACTTGTTGGCGAGA) [3], and after amplification using the primers tRNA_GLN_-F: 5′-AGGCATGCTGGGGAGGTACCGGTTCCATGGTGTAATGGTT-3′ and Scaf-ITR-R: 5′-CTAGGGGTTCCTGCGGCCGCAAAAATCTCGCCAACAAGTTG-3′, were cloned using the ClonExpress® II One Step Cloning kit (Vazyme Biotech, China) to generate pX601-CMV:SaCas9-U6:sgRNA, which was digested between the *Kpn*I and *Not*I sites. The constructed vector was then transformed into DH5α competent cells and transformants containing pX601-CMV:SaCas9-tRNA:sgRNA were identified by sequencing and, following subculturing, DNA was extracted. The EF1α promoter was amplified from pLentiCRISPR V2 vector using primers EF1a-F: 5′-CCTGCGGCCTCTAGACTCGAGGTGGGCAGAGCGCACATCGC-3′ and EF1α-R: 5′-TGGGGCCATGGTGGCACCGGTCCTGTGTTCTGGCGGCAAAC-3′. Then, pX601-CMV:SaCas9-tRNA:sgRNA, digested between the *Xho*I and *Age*I sites immediately before the SaCas9 gene, and the promoter were cloned using the ClonExpress® II One Step Cloning kit. Vector transformation, identification and plasmid extraction methods were performed as described above, to obtain pX601-EF1α:SaCas9-tRNA:sgRNA. The proposed structures of the two vectors, pX601-CMV:SaCas9-U6:sgRNA and pX601-EF1α:SaCas9-tRNA:sgRNA, are shown as Fig. 1a. The complementary oligonucleotides sgMstn1 and sgMstn2 were annealed for form a double-strand and were ligated to *Bbs*I-digested pX601-CMV:SaCas9-U6:sgRNA and pX601-EF1α:SaCas9-tRNA:sgRNA. Finally, the plasmids pX601-CMV:SaCas9-U6:sgRNA1, pX601-CMV:SaCas9-U6:sgRNA2, pX601-EF1α:SaCas9-tRNA:sgRNA1 and pX601-EF1α:SaCas9-tRNA:sgRNA2 (abbreviated as psgMstn1, psgMstn2, RpsgMstn1 and RpsgMstn2) were constructed.

### Cell culture and transfection

A total of 5 × 10^5^ C2C12 cells were seeded into 6-well plates in 2 ml DMEM supplemented with 10% FBS. After 24 h, the C2C12 cells were cultured to 50%–60% confluence and then transfected with psgMstn1/psgMstn2/RpsgMstn1/RpsgMstn2 and 1×PEI (polyethylenimine transfection reagent). Each plasmid was transfected into 5 wells, and there were 5 control wells that were not transfected. All these cells were incubated at 37 °C in a CO_2_ incubator for 48 h and harvested. The cells were subjected to genomic DNA extraction and western blotting experiments.

### AAV vectors for the CRISPR/Cas9 system

Procedures were carried out as previously described [14]. For the production of rAAV9–SaCas9 viruses, HEK293 cells were seeded into 10 dishes (100 × 10 mm; 5 × 10^6^ cells per dish). After 48 h, the cells were triple-transfected with pX601/psgMstn2/RpsgMstn2, pAAV9-RC and phelper according to the instructions provided with 1×PEI transfection reagent. After 72 h, the cells were harvested by scraping into media, centrifuged at 1000 × *g* for 10 min and resuspended in 1 ml of 1×PBS (phosphate-buffered saline). The cell suspension was subjected to three freeze–thaw cycles at −80 °C and at 37 °C. After fast centrifugation and filtration, the cell debris was cleared. The viral solution was concentrated by loading onto a density gradient column. After one round of ultracentrifugation, the high-density viruses were separated and extracted, and ran through dialysis bags for desalting [15]. The titers of the purified rAAV9–SaCas9 viruses were determined using the RT-PCR-based method described previously [16]. pX601 was diluted from 10^9^ copies/μl to 10^3^ copies/μl as the standard solution. The primers were: ITR-QPCR-F: 5ʹ-CGGCCTCAGTGAGCGA-3ʹ and ITR-QPCR-R: 5ʹ-AGGAACCCCTAGTGATG-3ʹ. The resulting viruses were designated AAV-CMV:SaCas9-U6:sgRNA, AAV-CMV:SaCas9-U6:sgRNA2 and AAV-EF1α:SaCas9-tRNA:sgRNA2 (abbreviated to AAV-CN, AAV-sgMstn2 and RAAV-sgMstn2, respectively).

### Animal experiments

Animal experiments were approved by the Animal Research Committee of Henan Agricultural University and all animals were maintained in a specific pathogen-free animal facility according to the related ethical regulations instilled at Henan Agricultural University and the guide for the care and use of laboratory animals.

### Mice

Forty-five C57BL/10 male mice in SPF grade (12 months of age) were purchased from the Center of Experimental Animal of Guangdong province (Guangzhou, China). Then, these mice were fed to the age of 18 months and were healthy without any abnormalities. The average weight of these mice was weighted about 33.5 g and 30 mice in the weight range of 33.5 ± 1 g were selected from the 45 mice for the experiment.

### Recombinant AAV transduction in vivo

**Fig. 2:**
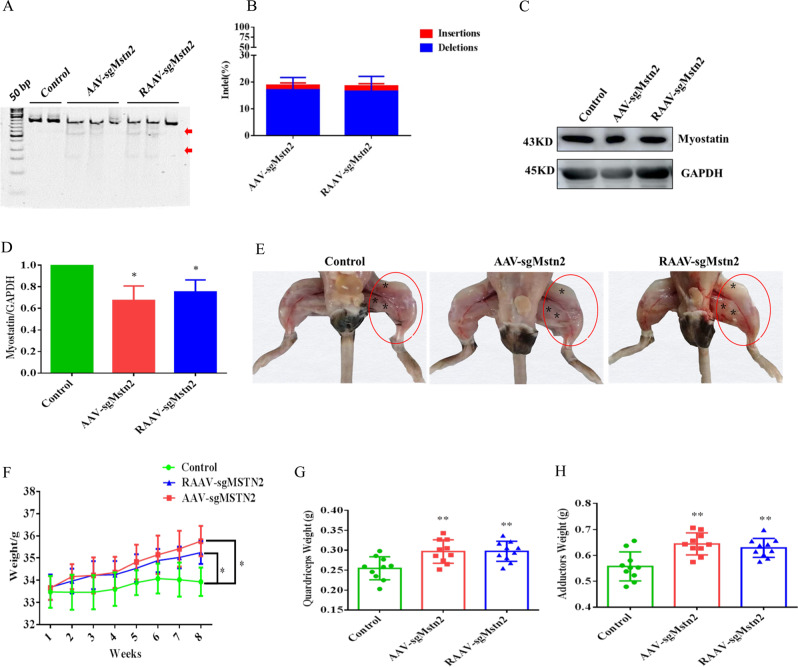
AAV-delivery of SaCas9 for genome editing in the left thigh of aged C57BL/10 male mice. **a** T7EI assay of mice genes produced by intramuscular injections of rAAV–SaCas9. Numbers of mutant muscle tissues with bands of 168, 338 and 506 bp in AAV-sgMstn2, RAAV-sgMstn2 and only one band of 506 bp in AAV-CN. The red arrow indicates the band of enzyme digestion. **b** Stacked histogram showing the percentage distribution of indels at *Mstn* in muscle tissues of mice, as measured by sequencing analyses. Data are presented as mean ± SEM from five mice in each group. **c** The muscle tissues in AAV-sgMstn2, RAAV-sgMstn2 and AAV-CN groups analyzed by western blot using an anti-MSTN antibody. And anti-GAPDH was used as loading control. **d** Stacked histogram showing the percentage distribution of MSTN expression in different muscle tissues. Data represent means ± SEM (The number of samples was 3 per group, and each sample was repeated three times). *: 0.01 < *P* < 0.05, **P < 0.01 when AAV-sgMstn2, RAAV-sgMstn2-treated were compared with AAV-CN-treated. **e** Changes of left thigh muscle mass in the aged mice in AAV-CN, AAV-sgMstn2 and RAAV-sgMstn2 groups. Compared with the AAV-CN-treated group, the muscle mass of the MSTN gene knockout group was significantly increased by AAV-sgMstn2 and RAAV-sgMstn2-treated groups (in red cycle). **f** Changes in body weight from one to eight weeks in different experimental groups. Data represent means ± SEM, *N* = 10 per group. **g**, **h** The weight of the quadriceps and adductor muscle in the different groups at 8 week. Data represent means ± SEM, *N* = 10 per group. *: 0.01 < *P* < 0.05, **P < 0.01, when AAV-sgMstn2, RAAV-sgMstn2-treated were compared with AAV-CN-treated.

To transduct skeletal muscle in vivo, the 30 male C57BL/10 aged mice used in the experiment were randomly divided into three groups, every group of 10 mice were injected with AAV-CN, AAV-sgMstn2 or RAAV-sgMstn2, respectively. Three points on the quadriceps and adductor muscles in the left thigh of the aged mice (10/group) were injected with ~2 × 10^11^ vg of AAV-sgMstn2/ RAAV-sgMstn2 in 100 µl of PBS. 10 control aged mice were treated similarly with ~2 × 10^11^ vg of AAV-CN. The injection sites were marked with asterisk in Fig. 2e. After 8 weeks, all the quadriceps and adductor muscles were collected for genomic DNA extraction, RNA extraction, western blotting and tissue slice experiments.

### T7 endonuclease 1 (T7E1) cleavage assay and targeted deep-sequencing analysis

Genomic DNA was extracted with the Tissue and Cell Culture DNA Midi kit (TianGen, Beijing, China) according to the manufacturer’s instructions. The purified genomic DNA was used as a template to amplify a fragment of the *Mstn* gene using specific primers: MSTN-Test1-F: 5ʹ-GACAACTTCTGCCAAGAGCG-3ʹ and MSTN-Test1-R: 5ʹ-GGGAAACTAGTGCACATTTTAGACA-3ʹ, and MSTN-Test2-F: 5ʹ-TGTTTTGATGGGGCCTGATGT-3ʹ and MSTN-Test2-R: 5ʹ-TGGAGTAAGATACTTTGTCTGGC-3ʹ. The fragment sizes amplified by these primer sets were 521 and 548 bp, respectively. The PCR products were digested with T7 endonuclease 1 (NEB, Massachusetts, USA) and resolved by 1.5% agarose gel electrophoresis. The tracking of indels by decomposition (TIDE) primers were the same as the MSTN-Test-F/R primers. Genomic DNA (100 ng) was used for PCR amplification with High Fidelity 2× PCR Master Mix (NEB, Massachusetts, USA). For TIDE analysis, 200 ng of PCR product was purified using the QIAquick PCR Purification Kit (Qiagen, Hilden, Mannheim, Germany) and sent for Sanger sequencing using the forward primer MSTN-Test-F. Insertion-deletion (indel) values were obtained using the TIDE web tool (https://tide.deskgen.com/) as described previously [17]. Targeted deep-sequencing analysis was performed for C2C12 cells and gDNA from mouse muscle using the PCR amplification approach. Briefly, the off-target locus was found through this website (http://www.rgenome.net/cas-offinder/), on-target or off-target locus-specific primers (Table 1) were used to amplify the editing site with Phusion High Fidelity DNA Polymerase (NEB, Massachusetts, USA). The resultant amplicons were separated on a 1.0% agarose gel. The bands of ~150 bp were extracted using the SanPrep DNA Gel Extraction kit. Then, targeted deep-sequencing of DNA products was performed by GENEWIZ Inc. In brief, the libraries were sequenced on an Illumina HiSeq (Illumina, California, USA) in paired-end mode with a read length of 150 bp. Primary analysis was performed using built-in software, HiSeq Control Software (HCS), RTA 2.3 plus, and demultiplexing was performed by bcl2fastq 2.17. Finally, the raw data of the targeted sequencing were analyzed by bioinformatics analysts at GENEWIZ Inc. The resulting indel frequencies, sizes and distributions were then plotted using GraphPad PRISM.

**Table 1:** List of off-target for sgRNA.

ID	Primer name	Sequence (5′ to 3′)	Site
mm4-intron-Dip2b-chr15	Mus-sg2-1offtarget-F	GTGGGCAGGACATGTGAGAA	GCAATGCGGAACAAGGTATACAACAGT
	Mus-sg2-1offtarget-R	ATGCTGTCGATTGCCTGGAA	
mm4-intron-Opa1-chr16	Mus-sg2-2offtarget-F	AGGCATTTTATAGATTCTGAGGTGA	GAAAGATGGTAAAAAGTAAACTTCAGT
	Mus-sg2-2offtarget-R	TCTTCCAGATGCTCAAGGGCT	
mm4-intron-Cntn5-chr9	Mus-sg2-3offtarget-F	CCTGTAGCTGATTGACTGGTAC	ACCTTTCTTTACCTAGTACTGTCTTTC
	Mus-sg2-3offtarget-R	GTGGGAAGGGGCGGTAAAAA	
mm4-intergenic-Gm26146-chr9	Mus-sg2-4offtarget-F	TGAGGGACACAGATGCCAAG	ACTGGAGTCTACATTTTACCGACTTTC
	Mus-sg2-4offtarget-R	GGTATGTGTGGGGAAAACCTCTT	
mm4-intron-Rybp-chr6	Mus-sg2-5offtarget-F	ACATTAAGTCTTAACTCAGGTGCT	GAAAGACAGTAAAGTGTATACTGGAAT
	Mus-sg2-5offtarget-R	TCCTCTTAGCCAGCTTACAGTA	
mm0-exon-Mstn-chr1	Mus-sg2-ontarget-F	CTCAGACCCGTCAAGACTCC	GAAAGACGGTACAAGGTATACTGGAAT
	Mus-sg2-ontarget-R	GCCTGGGCTCATGTCAAGT	
mm4-intergenic-St6galnac5-chr3	Mus-sg1-1offtarget-F	TGGGAGGTCTGGGGAATGTA	ACCATGTAGACATGGTGTTGATTGTCT
	Mus-sg1-1offtarget-R	AGACACCTGAGCACCCTACA	
mm4-intergenic-Gm22127-36789275	Mus-sg1-2offtarget-F	CTCAAAAGTCCAGTGGGCCA	AAAGAACCATTTCAATGCCTAAAGAGT
	Mus-sg1-2offtarget-R	TTCTGGTGAGACCCTCCCAA	
mm3-intergenic-Sema6a-chr18	Mus-sg1-3offtarget-F	GCTCTCTGCAGGCTATGTGA	ACACAATCTTTACCATGCCCAGATGGT
	Mus-sg1-3offtarget-R	CCCTGAACCATCCCACTGG	
mm4-intron-Pggt1b-chr18	Mus-sg1-4offtarget-F	TGCCCACTAGAATCAGCTGT	AAACAATGAATACGAAGCCTAGATAAT
	Mus-sg1-4offtarget-R	TGCCACCATGAACCAAGGTA	
mm4-intron-Klhl31-chr9	Mus-sg1-5offtarget-F	ACTGATATGTATGAGTGATACGCT	ATTACATGAGCTTTGTAATGATTGTTT
	Mus-sg1-5offtarget-R	AGCAAGCCCTAAGAATTTTCTTTGT	
mm4-intron-Cacna1e-chr1	Mus-sg1-6offtarget-F	ACCTCAGCGCATCCATATGG	AAACACTCATTTCCATGCAAATACAAT
	Mus-sg1-6offtarget-R	TGAAGCAGTTATGGAGTGGGT	
mm4-intergenic-P2ry1-chr3	Mus-sg1-7offtarget-F	AGGCACCCAGCTTTGTAGAA	AACCAATCATTTCCATTCCCAAAGGAT
	Mus-sg1-7offtarget-R	TCCACATGCAAGAGGTGACC	
mm0-exon-Mstn-chr1	Mus-sg1-ontarget-F	GGATGACAGCAGTGATGGCT	AAACAATCATTACCATGCCTACAGAGT
	Mus-sg1-ontarget-R	ACACTAGGACAGCAGTCAGC	

### RT-PCR analysis

Procedures were carried out as previously described [18]. RNA was isolated from mouse muscle using Trizol reagent (Invitrogen, Shanghai, China) as suggested by the manufacturer’s instructions. The RNA quality was determined by the ratio of absorbencies at 260 and 280 nm, while the concentration was determined by UV spectroscopy. cDNA was synthesized from 2 µg of RNA following the protocol supplied with the PrimeScript^™^ RT reagent kit (TaKaRa, Tokyo, Japan). Quantitative PCR (qPCR) was performed using SYBR Premix Ex Taq from TaKaRa. The qPCR reaction mixture contained 12.5 µl of the SYBR Premix Ex Taq (2×), 100 ng of cDNA and 0.5 µl of each forward and reverse primer (10 µM) in a total volume of 25 µl. The mixture was incubated at 95 °C for 30 s, followed by 40 cycles of 95 °C for 5 s and 60 °C for 30 s. The gene amplification values were normalized to GAPDH mRNA. The relative mRNA abundance was quantified using the comparative cycle threshold method. The primers used for real-time PCR are shown in Table 2.

**Table 2:** List of primers for real-time PCR.

Gene name	Primer sequence (5ʹ to 3ʹ)
QPCR-Mus-P21-F	AGTGTGCCGTTGTCTCTTCG
QPCR-Mus-P21-R	TCCCAGACAAAGTTGCCCTC
QPCR-Mus-CDK2-F	GGATTTCAGCCAAAGCAGCC
QPCR-Mus-CDK2-R	CAAGTCAGACCACGGGTGAA
QPCR-Mus-Smad2-F	CAGGACGGTTAGATGAGCTTGAGA
QPCR-Mus-Smad2-R	CCCACTGATCTACCGTATTTGCTG
QPCR-Mus-Akt-F	CATGAGGATCAGCTCGAACAGC
QPCR-Mus-Akt-R	ACGGGCACATCAAGATAACGG
QPCR-Mus-mTOR-F	CATTCATTGGAGACGGTTTG
QPCR-Mus-mTOR-R	TGAGAGAAATCCCGACCAGT
QPCR-Mus-FoxO1-F	AAGAGCGTGCCCTACTTCAA
QPCR-Mus-FoxO1-R	CTCTTGCCCAGACTGGAGAG
QPCR-Mus-GAPDH-F	TTCAACGGCACAGTCAAGG
QPCR-Mus-GAPDH-R	GCCTCACCCCATTTGATGTT

### Western blot analysis

Procedures were carried out as previously described [19]. Protein extracts were prepared on ice by homogenizing pieces of frozen tissues or cell pellets in 500 µl of RIPA Lysis Buffer (50 mM Tris-HCl, pH 8.0, 150 mM NaCl, 1% Triton X-100, 1% sodium deoxycholate, 0.1% SDS, 2 mM MgCl_2_) supplemented with 1:100 protease inhibitor solution (Roche, Basel, Swiss) by syringe blowing. With each loose- and tight-fitting piston, the samples received 30 strokes and were then centrifuged at 1000 × *g* for 5 min at 4 °C to remove debris. The supernatant (whole-cell lysate) was collected. For the isolation of membrane fractions, the supernatant was further centrifuged at 13,200 × *g* for 20 min at 4 °C, and the pellet was resuspended (2 µl/mg tissue) in sample buffer (2.7 M urea, 3.3% SDS, 0.167 M Tris pH 6.7). The protein concentration was estimated by a BCA assay. To detect protein, 30 µg of sample was separated by 10% SDS-PAGE and then transferred to polyvinylidene fluoride (PVDF) membrane. After incubation in 5% nonfat milk for 1 h, the membrane was incubated with rabbit polyclonal anti-Pax7/MyoD/MyoG/MuRF1/MSTN/GAPDH antibody (1:1000, Bioss Antibodies, Beijing, China, Catalog number: bs-22741R/bs-2442R/bs-3550R/bs-9391R/bs-23012R/bs-2188R) and mouse monoclonal anti-MAFbx/(1:500, ThermoFisher Scientific, Massachusetts, USA, Catalog number: PA5–91959) antibodies overnight at 4 °C. Then membranes were incubated with horseradish peroxidase-conjugated goat anti-rabbit (1:2000, Bioss Antibodies, Beijing, China, Catalog number: bs-0295G) and horseradish peroxidase-conjugated goat anti-mice (1:2000, Bioss Antibodies, Beijing, China, Catalog number: bs-0296G) antibodies for 1 h at room temperature. The target proteins were detected with the Luminata^™^ Crescendo immunoblotting HRP Substrate (Millipore, Massachusetts, USA).

### Histology and immunofluorescent analyses

The cross section of quadriceps muscles in the same position were fixed in para-formaldehyde and embedded in paraffin for further histopathological investigations. Formalin-fixed the quadriceps muscles sections were stained to investigate the density and diameter of muscle fibers by hematoxylin and eosin (H&E) according to a standard protocol [20]. After the staining procedure, the slides were scanned using a microscope. At least five fields at a ×200 magnification were randomly selected from each section in each group for imaging. The number of fibers was counted in each field, then converted to the number of fibers per mm^2^. Each section was analyzed to calculate the area of individual muscle fibers (mm^2^), by dividing the total area of muscle fibers (mm^2^) by the number of muscle fibers. All of the data were obtained and analyzed using Image Pro Plus 6.0 software. For immunostaining, Tissue-Tek OCT-embedded muscle tissues were snap-frozen in liquid nitrogen-cooled isopentane. To assess muscle pathology, 10 μm cryosections were cut and fixed in ice-cold acetone. Sections were stained as described previously [21]. Cross-section samples were immunostained with mouse monoclonal anti-SaCas9/anti-Pax7 primary antibody (1:200, Epigentek, California, USA, Catalog number: A-9001; 1:200, abcam, Cambs, UK, Catalog number:ab199010) and goat anti-mouse IgG-FITC/IgG-Alexa Fluor 647 (1:500, abcam, Cambs, UK, Catalog number:ab6785, ab150115) secondary antibody, and DAPI for nuclei. Muscle sections were imaged using standard fluorescence microscopy.

### Body weight of mice

The experimental mice were weighed on electronic scales. The body weights of the aged mice were recorded from weeks 1–8. For the measurement of muscle weight, quadriceps and adductor muscles from the left side of aged mice were dissected 2 months later. Ten aged male mice were weighed in each group.

### Statistical analyses

Data were expressed as the means ± SEM. Statistical significance was evaluated with one-way analysis of variance (ANOVA) for multiple groups comparisons using Prism 6 (GraphPad). *: 0.01 < *P* < 0.05, **P < 0.01.

## Results

### Efficient genome editing using all-in-one AAV–sgRNA–SaCas9 plasmids in cells and packaging all-in-one rAAV–SaCas9 viruses

Using pX601-CMV:SaCas9-U6:sgRNA, we generated an pX601-EF1α:SaCas9-tRNA:sgRNA construct containing SaCas9 under the expression of the EF1α promoter and with its sgRNA driven by the tRNA promoter (Fig. 1a) to exploit the small size of SaCas9. Two sites in the *Mstn* gene of mice are therapeutic targets for lowering age-related atrophy. These sites were selected initially to test the nuclease activity of SaCas9 in vitro (Fig. 1b). The psgMstn1, psgMstn2, RpsgMstn1 and RpsgMstn2 were transfected into mouse C2C12 cells. Gene editing was detected 3 days later via a T7 endonuclease assay. These vectors all showed noticeable editing of the *Mstn* gene (Fig. 1c). Using the TIDE web tool [22], on-target editing, the efficiencies of which at two target sites were evaluated by Sanger sequencing and indel quantification, was performed by sequence trace decomposition. We detected ~28.11% and 23.05% average indel values for the vectors psgMstn1 and RpsgMstn1, respectively, the majority of which were deletions, whereas psgMstn2 and RpsgMstn2 displayed high efficiency editing with ~31.48% and 30.09% average indel values (Fig. 1d). We also conducted western blot analysis of the transduced cell lysates. The results showed that the expression of MSTN protein was decreased by gene editing (Fig. 1e). Protein expression efficiencies were evaluated by grayscale analysis using Image J software, and the protein levels of psgMstn2 and RpsgMstn2 were less than those of psgMstn1 and RpsgMstn1 (Fig. 1f) (*P* < 0.01), and the off-target sites of psgMstn2 and RpsgMstn2 were fewer than those of psgMstn1 and RpsgMstn1. Therefore, we selected locus 2 as the subsequent packaging virus for the knockout of *Mstn* in vivo.

### The efficiency of *Mstn* knockout and the effect on muscle fiber quantity by intramuscular injection of rAAV–SaCas9 viruses in the left thigh muscle of aged C57BL/10 male mice

We tested the two SaCas9 systems in vivo. AAV-sgMstn2 and RAAV-sgMstn2 were produced and pseudotyped as serotype 9. A T7 endonuclease assay in muscle tissues showed obvious mutagenesis at the *Mstn* gene locus by both rAAV–SaCas9 viruses 8 weeks later. There was no evidence of mutagenesis in the control mice (Fig. 2a). On-target editing efficiencies at locus 2 were evaluated by Sanger sequencing. Indel quantification was conducted by sequence trace decomposition using the TIDE web tool. We detected ~20.81% average indel formation at the target site in muscle tissues receiving AAV-sgMstn2, which was around ~18.13% indel formation in muscle tissues receiving RAAV-sgMstn2 (Fig. 2b). Compared with the mice injected with AAV-CN, MSTN protein levels were substantially reduced in the left thigh muscle injected with AAV-sgMstn2 and RAAV-sgMstn2 (Fig. 2c, d) (*P* < 0.01). Efficient editing and MSTN protein reduction were signs of the success of the delivery and activity of rAAV–SaCas9 viruses at the *Mstn* locus. MSTN deficiency led to skeletal muscle hypertrophy in aged mice. Compared with the right thigh (the untreated thigh, as one of the few naturally paired organs, can also serve as an ideal control) or the mice injected with AAV-CN, muscle was substantially added in the left thigh of aged mice injected with AAV-sgMstn2 and RAAV-sgMstn2 (Fig. 2e). There was no great difference in body weight among the groups at the beginning of the experiment, whereas at the end of the experiment (especially in the last three weeks after injection), an increase was observed due to increased weight levels following injection with AAV-sgMstn2 and RAAV-sgMstn2 (Fig. 2f). Furthermore, we determined the therapeutic activity of AAV-sgMstn2 and RAAV-sgMstn2 on inhibiting muscle atrophy in aged mice. Treatment with AAV-sgMstn2 and RAAV-sgMstn2 significantly increased quadriceps weight (*P* < 0.01) and adductor weight (*P* < 0.01) compared with the AAV-CN-treated group (Fig. 2g, h). These results revealed muscle weight increases in the *Mstn* knockout-treated muscles.

### Effects on muscle-cell morphology of *Mstn* knockout in the left thigh muscle of aged C57BL/10 male mice

**Fig. 3:**
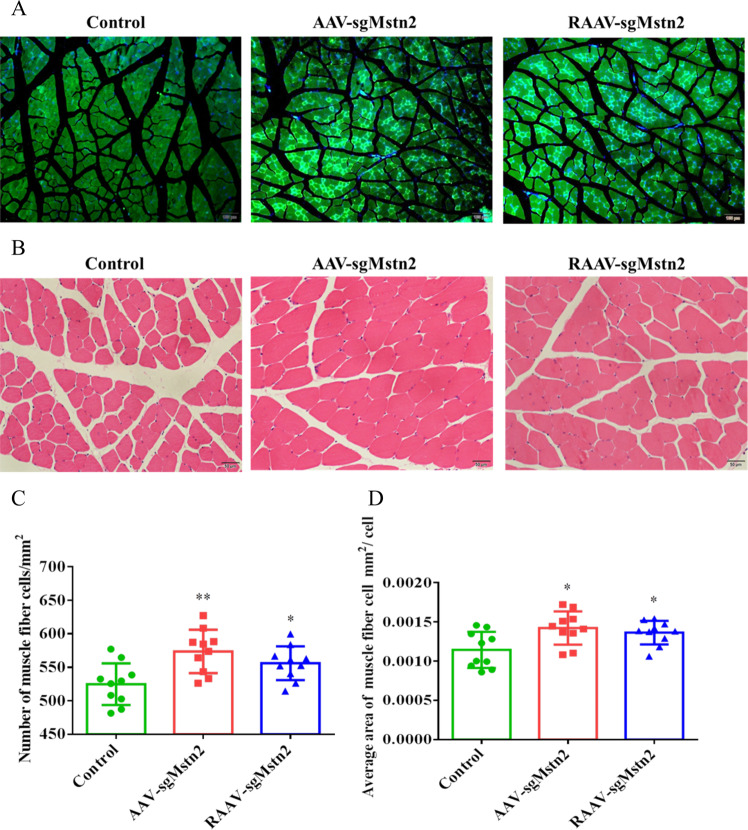
Changes in the physiological characteristics of muscle in the left thigh were achieved by intramuscular injection. **a** Tissue immunofluorescence test was performed on different groups of mice muscle sections (scale bar = 100 μm). An FITC fluorescence was observed for SaCas9 protein expression. Images depict cells of mice muscle in the left thigh for SaCas9 protein expression of rAAV. Similar fields of view also depict DAPI stained nuclei. **b** Sections were cut from each quadriceps muscle and stained with hematoxylin and eosin (H & E) so as to visually determine the extent of myofiber shape. Representative images of H & E stained with AAV-CN, AAV-sgMstn2, RAAV-sgMstn2-treated muscle sections are shown (scale bar = 50 μm). **c** The muscle fibers density as well as **d** the individual muscle fiber areas within each section was measured. Data represent means ± SEM, *N* = 10 per group. *: 0.01 < *P* < 0.05, **P < 0.01, when AAV-sgMstn2, RAAV-sgMstn2-treated were compared with AAV-CN-treated.

To elucidate the successful gene editing of mice muscles, we examined the expression of SaCas9 protein in AAV-sgMstn2 and RAAV-sgMstn2 in muscle cells. By immunostaining the muscle cells, we found green fluorescence in the nucleus of muscle sections of mice infected with the AAV-sgMstn2 and RAAV-sgMstn2 (Fig. 3a). H&E staining of the AAV-CN-treated and AAV-sgRNA2, RAAV-sgRNA2-treated quadriceps muscles was performed. In the same quadriceps muscle position, the transverse sections of the muscle fibers showed different density distributions. It was clear that the fiber areas differed in size (50 µm) when the transverse sections of the muscle fibers were enlarged (Fig. 3b). According to analysis of the quadriceps muscle sections, many new muscle fibers formed in the AAV-sgRNA2, RAAV-sgRNA2-treated muscles. The total number of muscle fibers increased in the experimental group and this change was significant when AAV-sgRNA2, RAAV-sgRNA2-treated are compared with AAV-CN-treated (Fig. 3c) (*P* < 0.01). Individual fiber areas were measured to assess fiber growth and thereby the progression of muscle growth. The average area of muscle fiber cells was significantly larger for the AAV-sgRNA2, RAAV-sgRNA2-treated muscles compared with those from the AAV-CN-treated group (Fig. 3d) (0.01 < *P* < 0.05). The results revealed that the number of muscle fibers increased and muscles enlarged with decreased MSTN expression in muscle tissues.

### MSTN deficiency activated the muscle-cell signaling pathway to allow muscle-cell improvement at the molecular level in aged mice

**Fig. 4:**
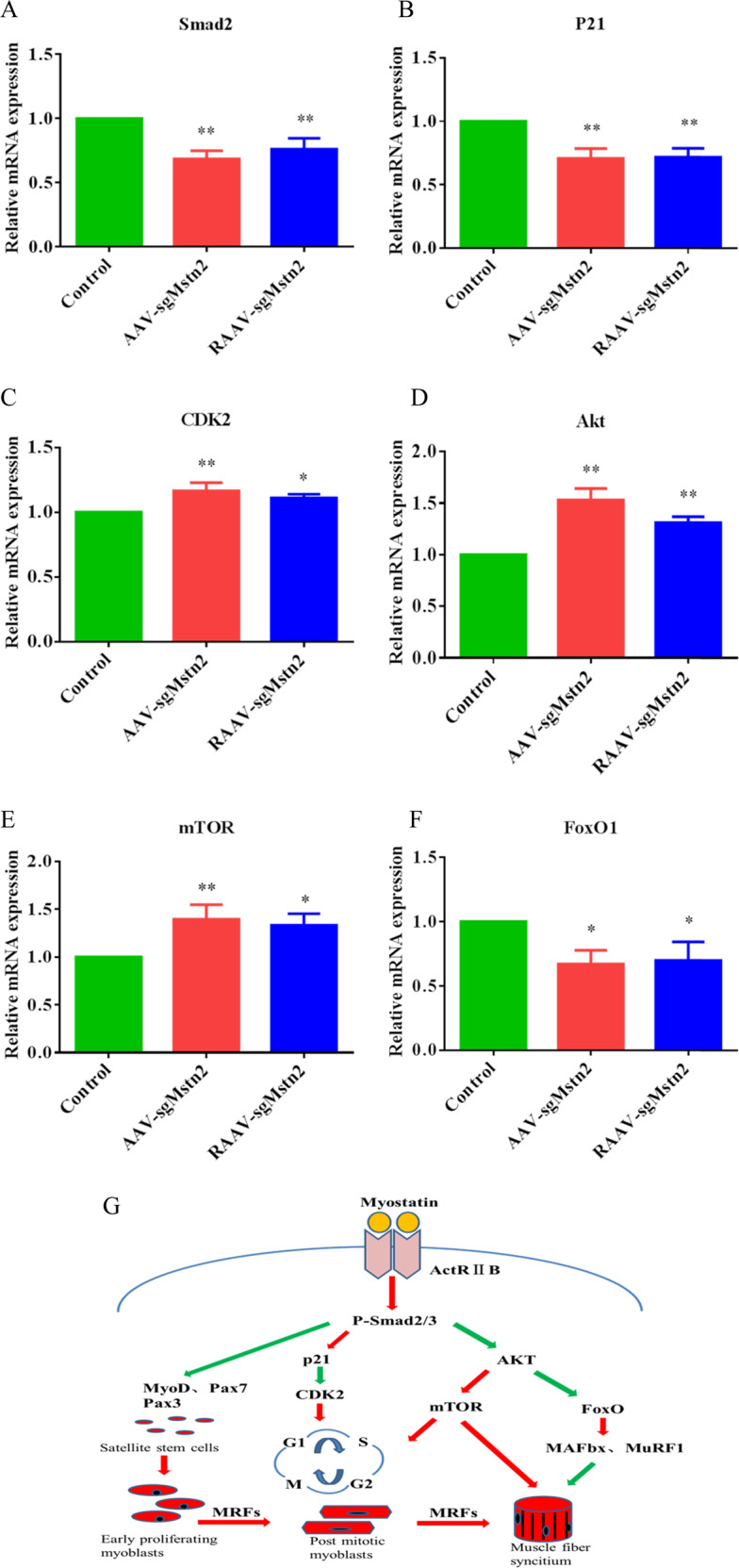
The cytokines expression were changed by AAV-CN, AAV-sgMstn2, RAAV-sgMstn2 treatment in muscle tissues. RT-PCR analysis was performed on 8 weeks on AAV-CN, AAV-sgMstn2, RAAV-sgMstn2-treated muscles in order to determine kinds of cytokines mRNA levels relative to GAPDH levels. Normalized data were used for generating the **a** Smad2, **b** p21, **c** CDK2, **d** AKT, **e** mTOR and **f** FoxO1 graphs depicted. Data represent means ± SEM (The number of samples was 3 per group, and each sample was repeated three times). *: 0.01 < P < 0.05, **P < 0.01 when AAV-sgMstn2, RAAV-sgMstn2-treated were compared with AAV-CN-treated. **g** A schematic were marked key cytokines of MSTN-related signaling pathway.

To confirm that the classic TGF-β signal pathway was involved in the observed improvement in muscle atrophy following knockout of *Mstn* in aged mice, we performed RT-PCR analysis of the cellular RNA in the quadriceps muscles. Smad2, p21 and CDK2 are important markers of the cell cycle of myoblasts. Phosphorylation of Smad2 activates the whole cell cycle. p21 and CDK2 levels signify the extent of myoblast proliferation as well as myoblast self-renewal. Experiments were performed to investigate Smad2, p21 and CDK2 mRNA levels in the quadriceps muscles of aged mice. The analysis of AAV-sgMstn2 and RAAV-sgMstn2-treated groups showed that Smad2 and p21 mRNA levels were lower than the AAV-CN-treated group (Fig. 4a, b) (*P* < 0.01). CDK2 levels, by contrast, were higher in the *Mstn* knockout groups than in the AAV-CN-treated group (Fig. 4c) (0.01 < *P* < 0.05). This showed that MSTN deficiency activated the cell cycle. To demonstrate the effect of *Mstn* knockout on the AKT/mTOR signal network of muscle cells, we also measured the expression of AKT, mTOR and FoxO1 in quadriceps muscles of aged mice. The AAV-sgMstn2 and RAAV-sgMstn2-treated groups showed increased expression of AKT in aged mice compared with the AAV-CN-treated group (Fig. 4d) (*P* < 0.01). The AAV-sgMstn2 and RAAV-sgMstn2-treated groups also showed increased expression of mTOR (Fig. 4e) (0.01 < *P* < 0.05). Furthermore, it was shown that the expression of FoxO1 was repressed by the AAV-sgMstn2 and RAAV-sgMstn2-treated groups (Fig. 4f) (0.01 < *P* < 0.05). The changes of these cytokines confirmed that the muscle cells were improved in the molecular level. These elucidated MSTN-related signaling pathways were simply plotted (Fig. 4g).

### MSTN deficiency promoted cell proliferation and differentiation by activating myogenic cytokines and reducing the apoptosis of muscles by inhibiting ubiquitin-proteasome ligases

**Fig. 5:**
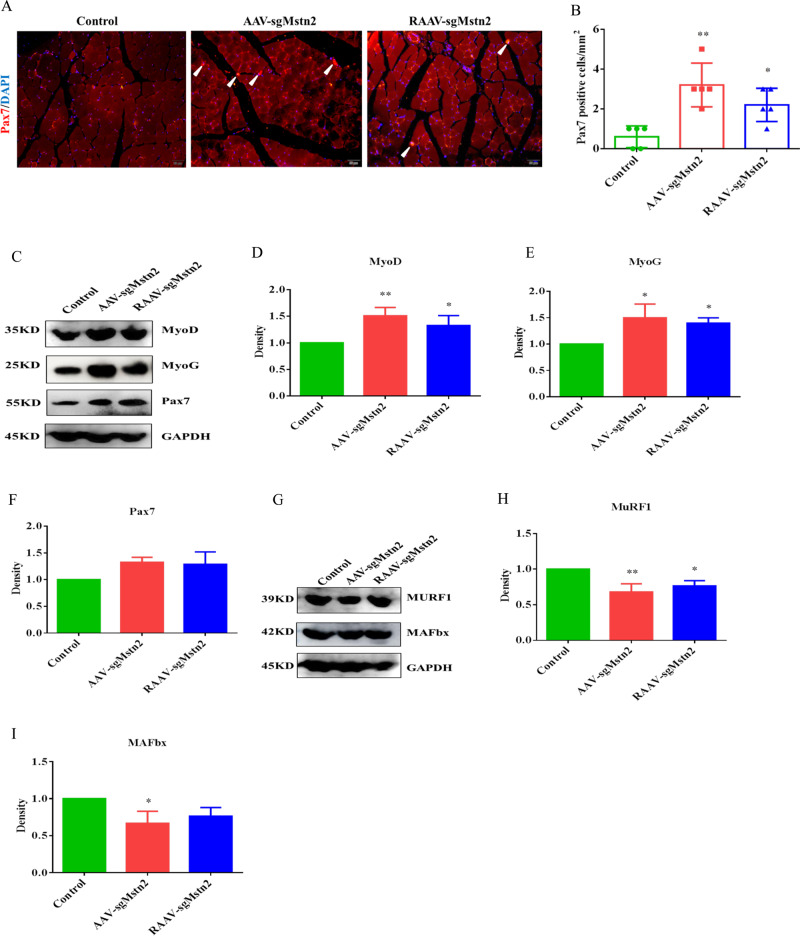
Satellite cells are increased in number and myogenic proteins changed during the *Mstn* knockout in the left thigh of aged C57BL/10 male mice. **a** Satellite cells expression. Immunofluorescent staining of muscle sections using a Pax7 antibody (in red). Representative images are shown. The nuclei were stained with DAPI (in blue). The white arrow indicates the satellite cell. **b** Quantification of the number of Pax7-positive cells/mm2 from **a**. Data are means ± SEM from five muscles derived from five different aged mice per group. *: 0.01 < *P* < 0.05, **P < 0.01. Western blot analysis was also performed in order to determine the kinds of cell factors protein levels. **c** MyoD, MyoG and Pax7 were detected during the *Mstn* knockout. Normalized data were used for generating the **d** MyoD, **e** MyoG and **f** Pax7 graphs depicted. Data represent means ± SEM (the number of samples was 3 per group, and each sample was repeated three times). *: 0.01 < *P* < 0.05, **P < 0.01 when AAV-sgMstn2, RAAV-sgMstn2-treated were compared with AAV-CN-treated. **g** Western blot was used to detect the effect of MSTN on the expression of MuRF1 and MAFbx proteins. Normalized data were used for generating the **h** MuRF1, **i** MAFbx graphs depicted. Data represent means ± SEM (the number of samples was 3 per group, and each sample was repeated three times). *: 0.01 < *P* < 0.05, **P < 0.01 when AAV-sgMstn2, RAAV-sgMstn2-treated were compared with AAV-CN-treated.

To demonstrate the mechanism responsible for the improvement in age-related atrophy in mice, we examined the number of satellite cells in the muscles of the AAV-sgMstn2, RAAV-sgMstn2 and AAV-CN-treated groups. By immunostaining for Pax7, a commonly used marker for satellite cells, we found a three-fold increase in the number of Pax7^+^ satellite cells on quadriceps muscle sections from the AAV-sgMstn2, RAAV-sgMstn2-treated groups compared with those from the AAV-CN-treated group (Fig. 5a, b). Previous studies have shown that MyoD resides in the satellite cells, interacts with MyoG and Pax7, and affects satellite cell activity and differentiation. Since MSTN down-regulated myogenic development was associated with MyoD, MyoG and Pax7, the expression levels of these proteins were detected by western blotting to confirm the changes in muscle mass. MyoD and MyoG were increased in expression in the AAV-sgMstn2, RAAV-sgMstn2-treated groups (Fig. 5c–e) (0.01 < *P* < 0.05). The Pax7 protein expression level were also increased in the AAV-sgMstn2, RAAV-sgMstn2-treated groups compared with the AAV-CN-treated group (Fig. 5c, f). To determine whether MSTN downregulation regulates muscle-cell apoptosis by affecting the ubiquitin-proteasome pathway, we performed western blotting to measure E3s protein levels. MuRF1 and MAFbx levels were lower in the AAV-sgMstn2, RAAV-sgMstn2-treated groups than in the AAV-CN-treated group (Fig. 5g–i) (0.01 < *P* < 0.05). This implied that MSTN downregulation suppresses the content of ubiquitin-protein ligases in aged mice, then promotes the levels of muscle-cell growth factor during myogenic development.

### SaCas9 is highly specific in vivo

**Fig. 6:**
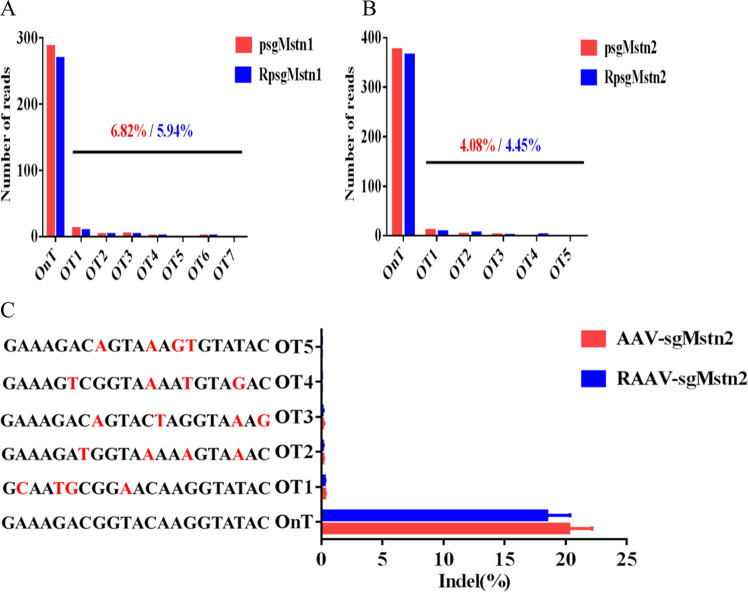
Targeted deep-sequencing of SaCas9. **a**, **b** Numbers of sequencing reads for the on-target (OnT) and off-target (OT) sites in C2C12 cells. **c** Targeted deep-sequencing to measure the muscle tissues of the OT sites. The mismatches of each OT site with the OnT protospacers is highlighted (red).

The possibility of off-target edits is a significant concern in therapeutic CRISPR/Cas9 genome editing. Some researchers have found that SaCas9 is a naturally high-occurring genome editing platform previous in mice [23, 24]. Using targeted deep-sequencing of mice cell, DNA products obtained by GENEWIZ Inc., we screened for off-target sites in the mice genome to determine whether SaCas9 maintains its minimal off-targeting profile in mice cells. C2C12 cells were transfected with psgMstn1, psgMstn2, RpsgMstn1 and RpsgMstn2 plasmids. The resulting genomic DNA was subjected to sequencing analysis. Sequencing revealed the read number of every off-target site in the mice genome. Seven potential off-target sites were identified for sgMstn1 and another five for sgMstn2, with a low read number probability of off-target sites in the mouse genome (Fig. 6a, b). We concluded that SaCas9 is intrinsically accurate and the sgRNA sites designed in the experiment is effective. We also performed targeted deep-sequencing using genomic DNA from the muscles of aged mice to validate these off-target probability. According to this more sensitive readout, indels were less detectable above background at all of the off-target locus 2 sites with *Mstn* (Fig. 6c). These results indicated that AAV-sgMstn2 and RAAV-sgMstn2 viruses are promising and safe candidates for in vivo applications.

## Discussion

The rapid development and application of CRISPR/Cas9 technology has led to a number of studies on its efficacy and specificity, although most of these studies have focused on SpCas9. Recent studies have shown that the relatively compact SaCas9 is active, mediating genome editing in vivo [25–27]. These studies showed that the efficiency of SaCas9 is comparable to that of SpCas9 using multiple assays and platforms. It has been found in three independent studies that SaCas9 is specific to different target sites in human cells [1, 28, 29]. By replacing several positively charged residues with uncharged amino acids, Zhang’s group developed a SaCas9 variant with higher specificity [1]. Here we demonstrated that two SaCas9 constructs were capable of gene disruption, and determined the genome editing efficiency of the two SaCas9 systems in vitro and in vivo. We generated two versions of SaCas9 constructs with different sgRNA promoters, U6 pro (~241 bp) and tRNA GLN pro (~72 bp), and different SaCas9 promoters, CMV pro (~584 bp) and EF1α pro (~212 bp), and found that the R-SaCas9 constructs (pX601-EF1α:SaCas9-tRNA:sgRNA) that were similar to SaCas9 (pX601-CMV:SaCas9-U6:sgRNA) could be harnessed to edit genomes and affect the expression of MSTN protein in C2C12 cells and in the muscle tissues of mice. These findings suggest that genetic therapy in vivo using SaCas9 constructs has the potential to be highly efficient.

To specifically study the effect of MSTN on muscle atrophy in aged mice, we also developed all-in-one AAV-sgMstn2 and RAAV-sgMstn2 viruses regulating MSTN. The single vector system is advantageous because an AAV virus contains both the SaCas9 and sgRNA. Therefore, only one virus is needed to mediate genome editing in animals or cell lines [30]. In general, we observed efficient genome editing with the two rAAV–SaCas9 viruses achieving ~20.81% and 18.13% genome mutation in the aged mice. Compared with other experiments, this study increased the amount of AAV virus injected by five times, and the editing efficiency was also higher [12, 31, 32]. These editing efficiencies are similar to those of a previous study examining SpCas9 genome editing in vivo [33]. Studies by Siriett and Wagner indicate that aging considerably influences myogenesis due to the muscle environment or systemic factors, such as the expression of myogenic regulatory factors and myoblast behavior. These experiments showed that the lack of MSTN significantly enhances muscle regeneration and reduces muscle atrophy [34, 35]. Furthermore, it was reported that the *Mstn* knockout could replicate this effect on muscle in aged mice. The body weight gain observed in anti-MSTN mice was significantly greater than that in control mice. Moreover, compared with control mice, the number and volume of muscle fibers were significantly increased in *Mstn* knockout mice. Schiaffino et al. [36] showed that inhibiting the MSTN protein promotes muscle hypertrophy and weight gain in adults. McPherron and Lee [37] knocked out the *Mstn* gene in mice and found that these mice had greater muscle mass, more widely distributed skeletal muscle groups and larger muscle fiber diameters. These findings are consistent with our experimental data. In our experiment, compared to the control group, the weight of the quadriceps and adductor muscles increased about 0.2–0.3 g and the total weight increased 1–1.5 g in *Mstn* knockout mice. We thought that the increased of total weight is not only the increased of local muscle mass, but also the slowed atrophy of muscle tissue, the increased of bone quality and the secretion of some hormones due to reduced MSTN protein [38–40].

In TGF-β-related signaling pathways, anti-MSTN increases the expression of CDK2 by reducing the expression of p21, thereby activating the cell from G2 phase to M phase. Thomas et al. [41] found that MSTN inhibits the phosphorylation of Rb protein by increasing p21 and reducing CDK2, thereby inhibiting the proliferation of myoblast. Our results confirmed this, as we showed that knockout of *Mstn* resulted in a decrease in Smad2 and p21. The changes in the levels of CDK2 were increased in the *Mstn* knockout groups. Furthermore, muscle growth or atrophy primarily depends on the activity of the AKT/mTOR signaling pathway [42]. MSTN has an influence on this signaling pathway, and inhibiting *Mstn* gene expression induces muscle growth primarily by stimulating protein synthesis through mTOR kinase and inhibiting degradation induced by FoxO transcription factors [43]. Our results showed that knockout of MSTN resulted in an increase in AKT and mTOR. The expression of FoxO1 was repressed by *Mstn* knockout in aged mice. These key cytokines had important effects on the anabolism of the muscle cells proliferation and differentiation related proteins and the occurrence of cell cycle.

Arnold and colleagues found that the downregulation of MSTN expression was accompanied by differential expression of myogenic regulatory factors. As a member of the myogenic regulatory factors, MyoG expression is closely related to skeletal muscle growth and differentiation. In this study, we showed that knockout of *Mstn* led to an increase in MyoD and MyoG in aged mice. Moreover, Pax7, a commonly used marker for satellite cells, was also increased in the *Mstn* knockout groups compared with the AAV-CN treatment group. This confirmed that the satellite cells were active and undergoing differentiation. Neither MAFbx nor MuRF1 is necessary for normal muscle growth, but their absence has been reported to reduce the rates of muscular atrophy [44]. During atrophy, MAFbx catalyzes the degradation of proteins that promote protein synthesis, while other ligases, especially MuRF1, catalyzes the ubiquitylation and degradation of myofibrillar components and the cytoskeleton [45, 46]. Our analysis of ubiquitin-protein ligases showed that MuRF1 and MAFbx levels were decreased in the *Mstn* knockout groups. These cytokines have a direct role in the proliferation, differentiation and atrophy of muscle cells.

Consitt and Clark [47] showed that MSTN signaling has an important role in inhibiting skeletal muscle growth, leading to metabolic dysfunction in the older population. Scimeca et al. [48] highlighted the role of the MSTN pathway in the physiological mechanism of sarcopenia in humans, highlighting anti-MSTN antibodies as a possible option for treating sarcopenia. Camporez and coworkers showed that the muscle mass and muscle strength of both young and old mice increased after treatment with anti-MSTN antibodies. This supports the pharmacological inhibition of MSTN as a potential therapy for age-related sarcopenia and metabolic diseases [49]. Our experiments also showed that reducing the expression of MSTN via the rAAV–SaCa9 viruses effectively improved muscle atrophy in aged mice. Although the inhibition of MSTN has not yet been applied in the treatment of sarcopenia diseases, MSTN and AAV–Cas9 are receiving more attention as potential therapeutic targets in this context.

In summary, we generated constructs with AAV-sgMstn2 and RAAV-sgMstn2 viruses targeting the *Mstn* gene. We thereby demonstrated that the two rAAV–SaCas9 viruses can be used to edit genomic DNA efficiently in aged mice. Through the efficient and rapid editing of *Mstn* in mice quadriceps muscle following intramuscular injection, changes to the number and area of muscle fiber cells and the proliferation and differentiation of satellite cells were observed. Furthermore, we verified the effect of *Mstn* knockout on atrophic muscle cells due to changes in the TGF-β signaling pathway and the ubiquitin-proteasome pathway. Both rAAV–SaCas9 viruses consistently showed that editing of the *Mstn* gene activated skeletal muscle proliferation and inhibited muscular atrophy in aged mice. Further studies may enhance our ability to use SaCas9 in research, broaden its applications in other fields and facilitate the potential clinical implementations of CRISPR/Cas9.

## Supplementary information

Article processing charge form

Supplementary information
