# Supplementary material for: Improvement of muscular atrophy by AAV–SaCas9-mediated myostatin gene editing in aged mice
Source: Cancer Gene Ther. 2020 May 13;27(12):960–75. doi: 10.1038/s41417-020-0178-7 (PMC7725670; doi:10.1038/s41417-020-0178-7)
Supplement: Supplementary file 1 — Article processing charge form [file 41417_2020_178_MOESM1_ESM.pdf]

LICENCE TO PUBLISH – OPEN ACCESS

SPRINGER NATURE

Manuscript Number: CGT-20-0061

Journal Name: Cancer Gene Therapy (the "Journal")

Proposed Title of the Article: Improvement of Muscular Atrophy by AAV-SaCas9-mediated Myostatin Gene Editing in Aged Mice (the "Article")

Author(s) [Please list all named authors, continuing on a separate sheet if necessary]: Shaoting Weng, Feng Gao, Juan Wang, Xingyu Li, Beibei Chu, Jiang Wang, Guoyu Yang (the "Author(s)")

Miscellaneous [for office use only]:

Licence applicable to the Article:
Creative Commons licence CC BY: This licence allows readers to copy, distribute and transmit the Article as long as it is attributed back to the author. Readers are permitted to alter, transform or build upon the Article, and to use the Article for commercial purposes. Please read the full licence for further details at - http://creativecommons.org/licenses/by/4.0/

will consider publishing this article, including any supplementary information and graphic elements therein (e.g. illustrations, charts, moving images) (the 'Article'), including granting readers rights to use the Article on an open access basis under the terms of the stated Creative Commons licence. Headings are for convenience only.

2 Grant of Rights
Subject to editorial acceptance of the Article, it will be published under the Creative Commons licence shown above. In consideration of the Licensee evaluating the Article for publication, the Author(s) grant the Licensee a non-exclusive, irrevocable and sub-licensable right, unlimited in time and territory, to copy-edit, reproduce, publish, distribute, transmit, make available and store the Article, including abstracts thereof, in all forms of media of expression now known or developed in the future, including pre- and reprints, translations, photographic reproductions and extensions. Furthermore, to enable additional publishing services, such as promotion of the Article, the Author(s) grant the Licensee the right to use the Article (including any graphic elements on a stand-alone basis) in whole or in part in electronic form, such as for display in databases or data networks (e.g. the Internet), or for print or download to stationary or portable devices. This includes interactive and multimedia use as well as posting the Article in full or in part on social media, and the right to alter the Article to the extent necessary for such use. Author(s) grant to Licensee the right to re-license Article metadata without restriction, including but not limited to author name, title, abstract, citation, references, keywords and any additional information as determined by Licensee.

3 Copyright
Ownership of copyright in the Article shall vest in the Author(s). When reproducing the Article or extracts from it, the Author(s) acknowledge and reference first publication in the Journal.

4 Self-Archiving
The rights and licensing terms applicable to the version of the Article as published by the Licensee are set out in sections 2 and 3 above. The following applies to versions of the Article preceding publication by the Licensee and/or copyediting and typesetting by the Licensee. Author(s) are permitted to self-archive a pre-print and an Author's accepted manuscript version of their Article.
a) A pre-print is the Author's version of the Article before peer-review has taken place ("Pre-Print"). Prior to acceptance for publication, Author(s) retain the right to make a Pre-Print of their Article available on any of the following: their own personal, self-maintained website; a legally compliant pre-print server such as but not limited to arXiv and bioRxiv. Once the Article has been published, the Author(s) should update the acknowledgement and provide a link to the definitive version on the publisher's website: "This is a pre-print of an article published in [insert journal title]. The final authenticated version is available online at: https://doi.org/[insert DOI]".
b) An Author's Accepted Manuscript (AAM) is the version accepted for publication in a journal following peer review but prior to copyediting and typesetting. Author(s) retain the right to make an AAM of their Article available on any of the following, provided that they are not made publicly available until after first publication: their own personal, self-maintained website; their employer's internal website; their institutional and/or funder repositories. AAMs may be deposited in such repositories on acceptance, provided that they are not made publicly available until after first publication. An acknowledgement in the following form should be included, together with a link to the published version on the publisher's website: "This is a post-peer-review, pre-copyedit version of an article published in [insert journal title]. The final authenticated version is available online at: http://dx.doi.org/[insert DOI]".

5 Warranties
The Author(s) warrant and represent that:
a) (i) the Author(s) are the sole copyright owners or have been authorised by any additional copyright owner to grant the rights defined in clause 2, (ii) the Article does not infringe any intellectual property rights (including without limitation copyright, database rights or trade mark rights) or other third party rights and no licence from or payments to a third party are required to publish the Article, (iii) the Article has not been previously published or licensed, (iv) if the Article contains materials from other sources (e.g. illustrations, tables, text quotations), Author(s) have obtained written permissions to the extent necessary from the copyright holder(s), to license to the Licensee the same rights as set out in clause 2 and have cited any such materials correctly;
b) all of the facts contained in the Article are according to the current body of science true and accurate;
c) nothing in the Article is obscene, defamatory, violates any right of privacy or publicity, infringes any other human, personal or other rights of any person or entity or is otherwise unlawful and that informed consent to publish has been obtained for all research participants;
d) nothing in the Article infringes any duty of confidentiality which any of the Author(s) might owe to anyone else or violates any contract, express or implied, of any of the Author(s). All of the institutions in which work recorded in the Article was created or carried out have authorised and approved such research and publication; and
e) the signatory (the Author or the employer) who has signed this agreement has full right, power and authority to enter into this Agreement on behalf of all of the Author(s).

6 Cooperation
a) The Author(s) shall cooperate fully with the Licensee in relation to any legal action that might arise from the publication of the Article, and the Author(s) shall give the Licensee access at reasonable times to any relevant accounts, documents and records within the power or control of the Author(s). The Author(s) agree that the distributing entity is intended to have the benefit of and shall have the right to enforce the terms of this agreement.
b) The Author(s) authorise the Licensee to take such steps as it considers necessary at its own expense in the Author(s)' name and on their behalf if the Licensee believes that a third party is infringing or is likely to infringe copyright in the Article including but not limited to initiating legal proceedings.

7 Author List
After signing, changes of authorship or the order of the authors listed will not be accepted unless formally approved in writing by the Licensee.

8 Edits & Corrections
The Author(s) agree(s) that the Licensee may retract the Article or publish a correction or other notice in relation to the Article if the Licensee considers in its reasonable opinion that such actions are appropriate from a legal, editorial or research integrity perspective.

9. Governing Law

Signed for and on behalf of the Author(s): Guoyu Yang

(PLEASE NOTE, ONLY HANDWRITTEN SIGNATURES ARE ACCEPTED)

Print Name: Guoyu Yang

Date: 2020-04-10

Address: 218 Pingan Avenue, Henan Agricultural University, Jinshui District, Zhengzhou, China
